# Isolated Dissection of the Superior Mesenteric Artery

**DOI:** 10.5811/cpcem.2017.7.34224

**Published:** 2018-01-09

**Authors:** Stacey Barnes, Beth Kushner

**Affiliations:** St. Joseph’s Regional Medical Center, Department of Emergency Medicine, Paterson, New Jersey

## Abstract

Isolated dissection of the superior mesenteric artery is a novel disease often presenting with vague signs and symptoms. Although the disease entity is rare, the potential for morbidity and mortality is high. This is a case report of a healthy 58-year-old male presenting with diffuse persistent abdominal pain. Diagnosed on computed tomography, this patient’s condition was managed conservatively with anticoagulants.

## INTRODUCTION

Spontaneous isolated dissection of the superior mesenteric artery (SIDSMA) is an extremely rare condition and a difficult diagnosis to make from the emergency department (ED). SIDSMA is associated with a wide range of clinical presentations, ranging from asymptomatic incidental finding to acute catastrophic bowel ischemia or aneurysmal SMA rupture.^1^ While certain factors such as hypertension, connective tissue disorders, vasculitis, atherosclerosis and trauma to the aorta may predispose patients to this condition, in the majority of cases no risk factors can be found.^2^,^3^ Depending on the extent of the dissection and the patient’s symptoms, treatment options vary from conservative treatment with anticoagulation to endovascular stenting, or even surgery.^1^,^4^ SIDSMA is a novel disease that requires immediate treatment and cannot be missed as a potentially life-threatening diagnosis in the ED.

## CASE REPORT

A 58-year-old male with a past medical history of migraine headaches, nephrolithiasis, and appendectomy presented to the ED with non-radiating left upper quadrant abdominal pain for one week. The pain was constant with no aggravating or relieving factors. The patient denied fever, urinary symptoms, vomiting, or change in appetite. He reported having more bowel movements than usual but stated they were of normal consistency. The patient denied rectal bleeding, and denied use of tobacco. He had a normal outpatient colonoscopy five months prior.

On physical examination, the patient’s vital signs were blood pressure of 117/87 milliliters of mercury, pulse 73 beats per minute, respiratory rate 16 breaths per minute, temperature 97.3 degrees Fahrenheit, and pulse oximetry 99% on room air. On physical examination, the abdomen was soft and had moderate left upper quadrant tenderness without guarding or rebound. Skin was without ecchymosis, petechiae, or purpura. Musculoskeletal exam revealed no deformities or swelling of joints. The rest of the physical exam was unremarkable.

**Image 1:**
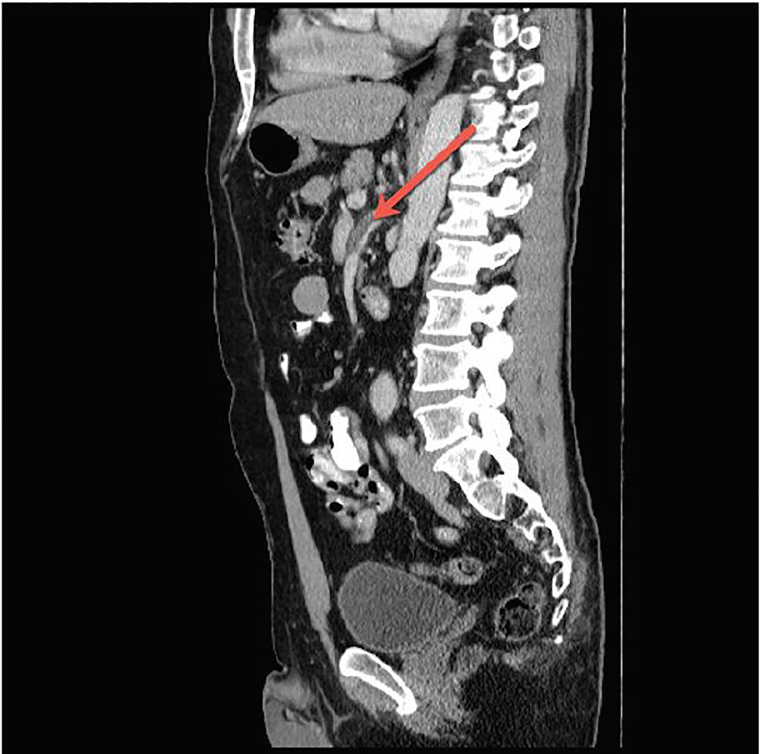
Sagittal computed tomography with intravenous contrast demonstrating thrombus (arrow) in the false lumen of the superior mesenteric artery dissection.

**Image 2:**
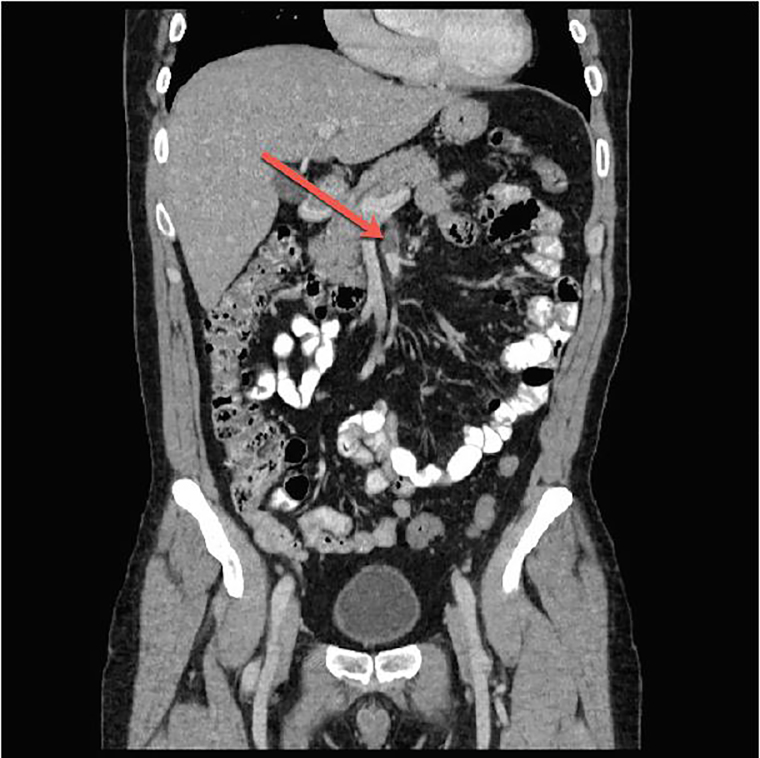
Coronal computed tomography with intravenous contrast demonstrating thrombus (arrow) in the false lumen of the superior mesenteric artery dissection.

Laboratory testing was ordered including a complete blood count, complete metabolic panel, lactic acid, and urinalysis that showed no acute abnormalities. The patient was initially treated with intravenous (IV) fluids and ketoralac during his ED stay. Computed tomography (CT) of the abdomen and pelvis was then completed with intravenous contrast. Results revealed aneurysm of the SMA with a four-centimeter dissection longitudinally along the course of the vessel (Images 1 and 2). Complicating the dissection was a 70–80% thrombosed lumen of a dissection flap that created a false lumen.

Vascular surgery was emergently consulted. As the patient was hemodynamically stable and the dissection with thrombosis did not reveal evidence of bowel ischemia, the patient was managed conservatively with IV heparin drip. During his hospital stay and repeat imaging, the patient did not show evidence of propagation of the dissection and the thrombus remained stable. He was converted to oral anticoagulation and discharged home with close follow-up.

## DISCUSSION

Isolated dissection of the SMA is a rare diagnosis made in the ED, but it is of critical clinical significance. Post-mortem findings of isolated SMA dissection suggest that if left undiagnosed and untreated, catastrophic compromise of intestinal blood supply can occur resulting in bowel ischemia and death.^5^ Spontaneous dissection of peripheral arteries is rare; however, after the carotid artery,the second most common peripheral artery to be affected is the SMA.^1^ The increase in reported incidence over the past several decades is likely secondary to the expanding use and higher-quality imaging capabilities of CT for undifferentiated abdominal pain.^2^,^3^ Unfortunately for the emergency physician, the three most common symptoms of SMA dissection are acute abdominal pain, abdominal pain with vomiting, and subacute intestinal obstruction. Interestingly, the fourth most common presentation for SMA dissection is an asymptomatic patient, with dissection found incidentally on CT.^4^ Acute abdominal pain is often reported as left upper quadrant and more rarely as postprandial pain, which is likely a sign of intestinal angina.^5^

Just as patient presentations are varied, so are the risk factors associated with SMA dissection. The majority of the cases reported are found in men 39–82 years old, with a mean age of 54. Smoking and hypertension appear to be a common factor in patients with SIDSMA. Unlike other forms of dissection, however, atherosclerosis is not a common risk factor. Other conditions such as previous abdominal surgery, diabetes or trauma have been reported with SMA dissection, but they have not been found to have a consistent correlation.^4^ Despite the variety of patients affected, the majority of the dissections begin in the retropancreatic portion of the SMA, which is most often in a fixed position.^6^ From this nidus, increased shearing forces lead to a tear in the intima or primary hemorrhage in the media itself. The mechanism is similar to an aortic transection at the ligamentum arteriosum seen in rapid deceleration injuries.^7^ Blood accumulates between the medial and adventitial layers or within the medial laminae leading to propagation of the dissection throughout the artery.^4^

CPC-EM Capsule

What do we already know about this clinical entity?

Spontaneous isolated dissection of the superior mesenteric artery is a rare condition and a difficult diagnosis to make from the Emergency Department as it has a variety of presenting symptoms.

What makes this presentation of disease reportable?

The presentation of this disease is reportable as it will increase awareness of emergency physicians to this rarity of this type of dissection, symptoms, and treatment.

What is the major learning point?

While nonspecific abdominal pain is common in the emergency department, it is vital for the physician to have a wide differential including this disease due to the high morbidity and mortality.

How might this improve emergency medicine practice?

By understanding the etiology and presentation of this fatal disease, the emergency physician will be more readily able to diagnose and treat superior mesenteric artery dissections.

As with other forms of dissection, CT performed with IV contrast in combination with advancement of multi-slice CT imaging techniques improves the accuracy in diagnosing both the dissection itself and the length of the dissection. In various other case reports, a seemingly otherwise normal CT only had increased fat attenuation around the SMA, which helped indicate to the practitioner that further investigation with angiography was necessary.^4^ Visualization of the intimal flap is considered a pathognomonic sign of isolated dissection; however, a mural thrombus is sometimes the only indication of a dissection.^8^

As indicated by the rarity of SMA, no standard therapeutic approach has been established. In decreasing frequency, treatment is separated into surgical, conservative, and endovascular approaches. The decision for route of management is directed by the symptoms the patient is experiencing. For patients such as the one described in this case, there were no signs of bowel ischemia or rupture of any of the branches from the SMA.^5^ In cases such as this, conservative management is often employed. Patients are started on a heparin drip and reimaged to ensure no further dissection has occurred.^7^ If repeat imaging is found to be stable, patients are then switched to oral anticoagulation and discharged home with close follow-up with vascular surgery.

A variation to conservative management includes the addition of an antiplatelet agent, which has been described in several case reports.^5^ Due to lack of clinical trials, there are great discrepancies for length of follow-up and optimal intervals for reimaging. Additional considerations in treatment involve whether or not a thrombus is present in the false lumen. With a thrombus present, patients are at increased risk of potential bowel ischemia and for this reason warfarin is continued on an outpatient basis. It should be emphasized that in previous case reports it has been noted that although conservative therapy does prevent new thrombus formation, progression of disease has occurred, resulting in the need for urgent endovascular or surgical intervention.^9^

For patients who have signs or symptoms of intestinal ischemia or impending aneurysm rupture, endovascular or surgical technique is preferred. Endovascular treatment includes stent placement, intralesional thrombolytic therapy, and balloon angioplasty.^6^,^9^ Surgical treatment includes aortomesenteric bypass, direct transposition of the SMA to the infrarenal aorta, or even patch angioplasty. Due to the invasive nature of surgery, operative intervention is often reserved for patients with bowel necrosis or increasing size of aneurysm despite medical management. Although endovascular treatment has been advocated recently and performed successfully several times per the case literature, there is still no consensus about which treatment modality is the gold standard for isolated SMA dissection.^9^

## CONCLUSION

Our case demonstrates that patients with persistent abdominal pain, even without standard risk factors for dissection of the carotid or aortic arteries, are still prime candidates for superior mesenteric artery dissection. With technological advances in CT,the practitioner is able to diagnose and halt the propagation of a potentially lethal disease. Patients whose symptoms are manageable and who are without signs of bowel ischemia or injury to vessels distal to the dissection can be treated conservatively with a heparin drip and converted to oral anticoagulants with close follow-up as an outpatient. Case reports of SMA dissection have been reported in surgical and gastroenterology literature, but are novel to emergency medicine. Detection and treatment of this challenging disease will increase and is something every emergency physician should be familiar with. While isolated dissection of the SMA is a rare disease, the potential for morbidity and mortality is high. Therefore, this is a critical diagnosis to make in the ED.
